# Patterns of illness and care over the 5 years following onset of psychosis in different ethnic groups; the GAP-5 study

**DOI:** 10.1007/s00127-017-1417-6

**Published:** 2017-07-05

**Authors:** Olesya Ajnakina, John Lally, Marta Di Forti, Anna Kolliakou, Poonam Gardner-Sood, Javier Lopez-Morinigo, Paola Dazzan, Carmine M. Pariante, Valeria Mondelli, James MacCabe, Anthony S. David, Fiona Gaughran, Robin M. Murray, Evangelos Vassos

**Affiliations:** 1 Department of Psychosis Studies, Institute of Psychiatry, Psychology and Neuroscience, King’s College London, 16 De Crespigny Park, London, SE5 8AF UK; 2 National Institute for Health Research (NIHR) Mental Health Biomedical Research Centre at South London and Maudsley NHS Foundation Trust and King’s College London, De Crespigny Park, London, SE5 8AF UK; 3 MRC Social, Genetic and Developmental Psychiatry Centre, Institute of Psychiatry, Psychology and Neuroscience, King’s College London, De Crespigny Park, London, SE5 8AF UK; 4 Department of Psychological Medicine, Institute of Psychiatry, Psychology and Neuroscience, King’s College London, 16 De Crespigny Park, London, SE5 8AF UK; 5 Department of Psychiatry, Royal College of Surgeons in Ireland, Beaumont Hospital, 123 St Stephen’s Green, Dublin 2, Ireland

**Keywords:** First episode psychosis, Ethnicity, Ethnic minorities, Longitudinal outcomes, Pattern of care, Social isolation, Clinical outcomes

## Abstract

**Purpose:**

Previous research has not provided us with a comprehensive picture of the longitudinal course of psychotic disorders in Black people living in Europe. We sought to investigate clinical outcomes and pattern of care in Black African and Black Caribbean groups compared with White British patients during the first 5 years after first contact with mental health services for psychosis.

**Methods:**

245 FEP cases aged 18–65 who presented to psychiatric services in 2005–2010 in South London (UK). Using the electronic psychiatric clinical notes in the South London and Maudsley NHS Foundation Trust (SLaM), extensive information was collected on three domains—clinical, social, and service use.

**Results:**

During the 5-year follow-up (mean = 5.1 years, s.d. = 2.4; 1251 person years) after first contact with mental health services, a higher proportion of Black African and Black Caribbean ethnicity had compulsory re-admissions (*χ* ^2^ = 17.34, *p* = 0.002) and instances of police involvement during an admission to a psychiatric unit (*χ* ^2^ = 22.82, *p* *<* 0.001) compared with White British ethnic group. Patients of Black African and Black Caribbean ethnicity did not differ from the ethnic group in overall functional disability and illness severity, or frequency of remission or recovery during the follow-up period. However, patients of Black ethnicity become increasing socially excluded as their illness progress.

**Conclusions:**

The longitudinal trajectory of psychosis in patients of Black ethnicity did not show greater clinical or functional deterioration than white patients. However, their course remains characterised by more compulsion, and longer periods of admission.

**Electronic supplementary material:**

The online version of this article (doi:10.1007/s00127-017-1417-6) contains supplementary material, which is available to authorised users.

## Introduction

Psychiatric epidemiology has consistently demonstrated that the incidence rates of psychotic disorders are considerably elevated among those of Black ethnicity residing in the UK compared to the host population [1–3]. The evidence further suggests that individuals of Black ethnicity are more likely to make the first contact with mental health services via admissions under Mental Health Act (MHA) legislation [4], in many cases with police present at admission [5, 6], or to be admitted to high-security psychiatric hospitals [7] compared to White British patients. There are some indications that this pattern of increased compulsory care persists over the course of their illness [8–10].

Over the past 20 years there has been an increased focus on specialist early intervention services for first episode psychosis (FEP) [11, 12] which ignited recognition that individuals with psychotic disorders can experience symptomatic improvements and regain a degree of social and occupational functioning [13]. The evidence is consistent that one-third of patients with FEP recover [14, 15]. Yet, it is still unclear whether this recovery rate applies to those in Black ethnic groups. Reports are mixed in relation to the symptomatic remission in Black populations with some reporting that remission is more common in Black ethnic groups [16], while others argue an opposite view [10]. Importantly, earlier research into longitudinal illness trajectory across ethnic groups is marked by methodological limitations, such as small sample sizes [8] and a tendency to neglect the diversity in culture, religious beliefs and life experience between Black African and Black Caribbean populations by combining these ethnic groups in analyses [8, 9, 16, 17]. Furthermore, some investigators have limited their sample to those with diagnosis of schizophrenia only [5, 17] or who had been re-admitted during a follow-up period, and as such bias results towards poorer outcomes [18].

Cumulatively, previous research has not provided us with a comprehensive picture of the true course of psychotic disorders in Black African and Black Caribbean ethnic groups, and whether the intensity of care delivered to Black ethnic groups reflects the severity of their psychopathology. Therefore, using a quasi-prospective cohort design and utilising the data from a large and well-characterised sample of patients with FEP, we sought to investigate clinical and social outcomes in Black African and Black Caribbean ethnic groups compared with White British patients. We further tested whether the intensity of care delivered to Black ethnic groups was reflected in their overall functional disability and illness severity in the illness course after first contact with mental health services for psychosis. Our null hypothesis was that the clinical course and pattern of care in patients of Black ethnicity would not be different from patients of White British ethnicity. As the evidence suggests that the first 3–5 years after first illness onset constitutes a critical period for intervention [19, 20], we focused on the first 5 years of illness after first contact with mental health services for psychosis.

## Methods

### Sample

Participants for this study were recruited as part of the National Institute of Health Research (NIHR) Biomedical Research Centre (BRC) Genetics and Psychosis (GAP) study conducted in South London, UK. Further details of the study are available in Di Forti et al. [21]. Briefly, the GAP study comprised individuals aged 18-65 years who presented to the psychiatric services of the South London and Maudsley (SLaM) National Health Service (NHS) Foundation Mental Health Trust between December 2005 and October 2010 with a first episode of psychosis (FEP) [International Classification of Diseases (ICD)-10; F20–F29 and F30–F33] [22], validated by administration of the Schedules for Clinical Assessment in Neuropsychiatry (SCAN) [23]. Cases were excluded if there was evidence of (1) psychotic symptoms precipitated by an organic cause; (2) transient psychotic symptoms resulting from acute intoxication as defined by ICD-10; (3) head injury causing clinically significant loss of consciousness; and (4) learning disability (IQ < 70).

### Ethics

The GAP study was granted ethical approval by the South London and Maudsley and Institute of Psychiatry Local Research Ethics Committee (reference number: 05/Q0706/158). All cases gave informed written consent after reading a detailed information sheet.

### Assessments at baseline

#### Socio-demographic characteristics

Information on socio-demographic characteristics was collected using a modified version of Medical Research Council (MRC) Socio-demographic Schedule [24]. Ethnicity was self-ascribed from the 16 categories employed by the UK Census in 2001 (http://www.statistics.gov.uk/census2001). Similar to a previous study [25], we categorised the ethnic groups as follows: (1) White British category that included all individuals of white ethnicity who were born in England, Scotland, Wales and Northern Ireland; (2) Black African category included all Black participants born in sub-Saharan Africa or born in the UK with at least one parent of sub-Saharan African origin; and (3) Black Caribbean category comprised all Black individuals born in the Caribbean or born in the UK with at least one parent of Caribbean origin. Patients of mixed Caribbean–African parentage and other ethnicities were excluded from the analysis.

#### Clinical assessments

Duration of untreated psychosis (DUP) was defined as the time between the date of onset of first psychotic symptoms to the date of treatment with antipsychotic medications [26, 27]. Age at first contact was defined as the age at which a patient came into contact with mental health services for the first time following onset of psychotic symptoms [16]. The baseline diagnoses were made from face-to-face interviews and mental health records according to ICD-10 criteria [28] utilising the Operational Criteria Checklists (OPCRIT) [29]. The OPCRIT system consists of a 90-item checklist and uses computerised diagnostic algorithms based on published criteria to provide a diagnostic category for each subject employing a number of classification systems [29]. All diagnoses were performed by qualified psychologists and psychiatrists, subject to comprehensive training and achievement of good inter-rater reliability (*κ* = 0.97). Similarly, qualified psychologists and psychiatrists completed Global Assessment of Functioning (GAF) scales from face-to-face interviews with good inter-rater reliability (*κ* = 0.90). GAF scored were used to measure both overall symptoms severity and functional disability associated with the illness at the study entry [30].

### Tracing procedure

Approximately 5 years after first contact with mental health services for psychosis, we sought to trace all cases who had given consent for follow-up and for their clinical records to be accessed. The follow-up data were extracted retrospectively using the electronic psychiatric clinical records (EPCRs). The EPCRs are the primary clinical records keeping system within the SLaM Trust that allows to search all clinical information, including correspondence, discharge letters and events, recorded throughout patients’ journeys through the SLaM Trust services [31]. All deaths and emigrations up to and including those that occurred during the final year of follow-up were identified by a case-tracing procedure with the Office for National Statistics (ONS) for England and Wales and the General Register Office (GRO) for Scotland.

### Data at follow-up

At follow-up, extensive information was extracted across clinical and social domains, and patterns of care, from electronic psychiatric clinical records using the WHO Life Chart Schedule (LCS) extended version [32]. This measure provides standardised retrospective assessments of patients’ experiences for the entire period of illness operationalised as the period from the first contact with mental health services for FEP to the date of the last assessment recorded in the electronic notes. The LCS has been shown to be reliable for follow-up assessments and adaptable across cultures [33].

#### Clinical

Similar to earlier work conducted in the same geographical region [14, 34] and in line with the operational criteria proposed by Andreasen et al. [35], administrative remission was operationalised as the absence of a clear record of psychotic symptoms in case notes for ≥6 months and was not dependent on absence of non-psychotic symptoms (e.g. depressed mood, neurotic manifestations), nor whether the patients were receiving a treatment with antipsychotic medications during remission. To define remission status, we examined the entire clinical record, including clinical notes recorded by treating clinicians, and correspondence relating to clinical assessments and clinical reviews, to document the clinical state of patients characterised by no psychotic symptoms for a continuous period at least 6 months or longer; this included no evidence of re-emergence of psychotic symptoms, re-admission to psychiatry wards, and/or having been re-referred to acute home treatment/crisis intervention services during the 6 month period. To be consistent with earlier studies [14], we defined recovery as sustained remission for ≥2 years. The duration of the baseline psychotic episode was defined as the period from the date of first contact with mental health services for a FEP to the date that the first 6-month period of remission started [14, 36]. That is the date that overt psychotic symptoms were first absent and thereafter did not return for at least 6 months. Similar to baseline, GAF was used to measure the overall illness severity and functional disability at the end of the 5-year follow-up period using the clinical notes. GAF scores collected from clinical records showed high comparability when compared to GAF scored collected from face-to-face interview (intra-class correlation = 0.81). To examine the deterioration of the overall functional disability and illness severity over the follow-up period, we deducted the baseline GAF scores from those obtained at the end of the follow-up period.

#### Patterns of care

Utilising the LCS extended version [32] and excluding hospital admission on first contact with mental health services for psychosis, we extracted detailed information on circumstances of each re-admission including all compulsory admissions [i.e., admissions occurring under mental health act (MHA) legislation] and instances when police were involved at the time of, or shortly before, hospital admissions throughout the 5-year follow-up period. Using the admission and discharge dates for each re-admission, we calculated the total length of inpatient admissions in psychiatric wards during the entire follow-up period. Time to first re-admission was defined as the time elapsed from first contact with psychiatric services for psychosis and the time to first re-admission. We further extracted a cumulative number of days of contact with community mental health services for all patients throughout the entire follow-up period.

#### Social

Using the LCS extended version [32], we extracted information on housing, employment, relationships and living arrangements from the electronic clinical records. We used these socio-demographic characteristics as markers indicative of the overall social functioning and integration at the end of the follow-up period.

### Analysis

We described primary outcomes using frequencies, percentages, mean and standard deviations, median and inter-quartile ranges (IQR). Between groups, comparisons were made using *χ* ^2^ tests for categorical variables; ANOVA tests, or Kruskal–Wallis tests, for continuous variables; rank *χ* ^2^ tests for count data. All analyses were two-tailed, and a *p* value ≤0.05 was considered statistically significant. All analyses were conducted in STATA release 14 (STATA Corp LP, USA).

## Results

### Sample

Within the study period, we approached 606 FEP patients; of these, 145 (24%) refused to participate. Thus, 461 patients with FEP cases were recruited to the original GAP study at baseline. The two most common reasons for refusal were lack of interest in the research and the length of study assessments. Patients who refused to participate were more likely to be men (*p* = 0.04) and of Black ethnic origin (*p* = 0·001) than were those who consented. The full information on socio-demographic characteristics at baseline was available for 449 (97.4% of 461) consented cases. Of these 152 (33.9% of 449 cases) patients were of either White Other (*n* = 58) or mixed/Asian (*n* = 94) ethnic background and thus were excluded from the analyses. Consequently, the baseline sample in the present study comprised 297 FEP patients. Of these, 111 (37.4%) were of White British, ethnicity, 110 (37.0%) were of Black African ethnicity and 76 (25.6%) were of Black Caribbean ethnicity. At the time of first contact with mental health for psychosis, a higher proportion of Black Caribbean patients lived alone (*χ* ^2^ = 6.98, *df* = 2, *p* = 0.03) and were unemployed (*χ* ^2^ = 7.24, *df* = 2, *p* = 0.03) compared to White British and Black African ethnic groups. There were no other differences between the ethnic groups at the time of first contact with mental health services for psychosis (Supplementary Table 1).

**Fig. 1:**
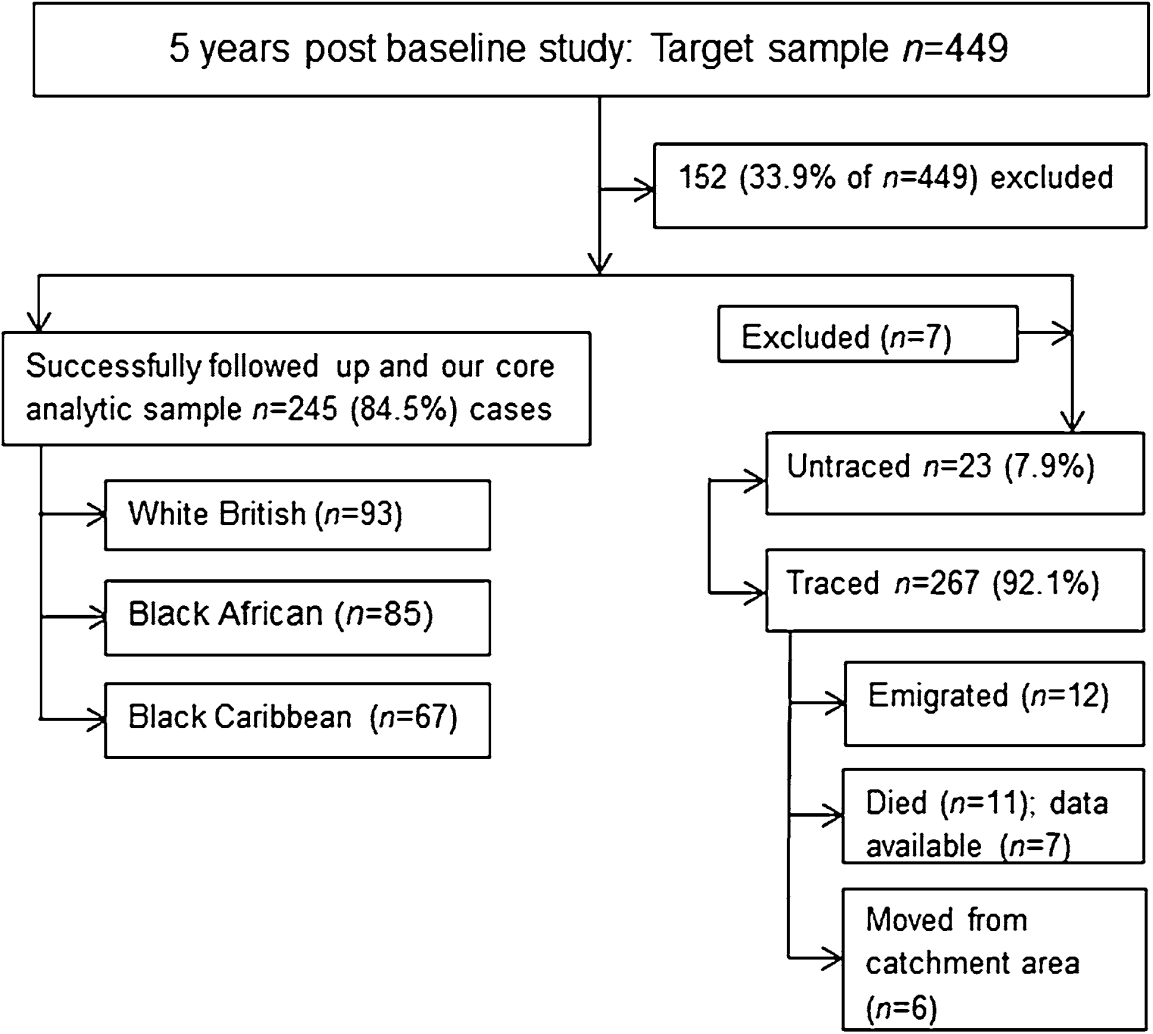
Demonstrates how cases were traced and administrative outcome

A flow chart depicting how the cases were traced and administrative outcomes is presented in Fig. 1. Approximately 5 years (mean_years_ = 5.1, s.d. = 2.4; 1251 person years) after first contact with mental health services, a total of 11 (3.7%) patients had died; but information on longitudinal outcomes was available for seven of these, thus these seven patients were included in all analyses. 12 (4.1%) patients had migrated, and six (2.1%) patients moved away from the catchment area. Additionally, seven (2.4%) patients were excluded as we did not have information on follow-up and their details were not available at baseline to enable us to trace them via ONS/GRO tracing procedures. We were unable to trace the whereabouts for 23 (7.9%) patients. Those patients who died during the follow-up period without any information on the course of their illness [*n* = 4 (1.4)] were older (mean_years_ = 44.5, s.d. = 18.4) (*F* = 4.05, *df* = 282, *p* = 0.003); and those who emigrated tended to be of Black African ethnicity (*χ* ^2^ = 18.36, *df* = 8, *p* = 0.02) (Supplementary Table 2). Cumulatively, we successfully traced 92.1% of our original sample and full information at follow-up was available for 84.5% (*n* = 245/290) of the cohort. FEP patients who were lost to follow-up were not different in the baseline characteristics from patients who had full follow-up data (Supplementary Table 3).

### Core analytic sample

Our core analytic sample comprised 245 (84.5% of *n* = 290) FEP patients with an average follow-up length of 5 years after first contact with mental health services for psychosis. This sample encompassed 93 (38.0%) patients of White British ethnicity, 85 (34.7%) patients of Black African ethnicity and 67 (27.3%) patients of Black Caribbean ethnicity. Patients of Black Caribbean ethnicity had the longest length of follow-up (mean_years_ = 5.6, s.d. = 2.6) compared to White British (mean_years_ = 4.9, s.d. = 2.4) and Black African (mean_years_ = 4.9, s.d. = 2.2) ethnic groups; though this difference did not meet the standard level for statistical significance (*F* = 2.12, *df* = 243, *p* = 0.12). 66% (158/240 cases) of the total sample were males, with Black African patients more likely to be male (*χ* ^2^ = 5.39, *df* = 2, *p* = 0.07) (Supplementary Table 4). Those with non-affective psychoses had significantly lower rates of remission and higher rates of repeated admissions and a higher proportion of homelessness at the end of the follow-up period (Supplementary Table 5). However, there was no difference in the proportions of patients of White British, Black African and Black Caribbean ethnicity between the affective and non-affective psychosis groups (*χ* ^2^ = 1.31, *df* = 2, *p* = 0.52).

### Clinical presentation over the follow-up period

Clinical illness course for the entire follow-up period after first contact with mental health services for psychosis by ethnicity is presented in Table 1. Over the 5-year follow-up period, 63.1% (*n* = 149/236) of the overall sample met criteria for remission and 28.4% (*n* = 63/222) met criteria for recovery at least once, with a median duration of the baseline episode of 8 weeks (IQR = 6–20). White British, Black African and Black Caribbean ethnic groups did not differ in these outcomes. During the follow-up period, no ethnic group showed a more rapid deterioration in overall illness severity and functional disability.

**Table 1:** Clinical outcomes over the first 5 years after first contact with mental health services for psychosis, by ethnicity

Clinical outcomes	Total (*n* = 245)	White British (*n* = 93) (38.0%)	Black African (*n* = 85) (34.7%)	Black Caribbean (*n* = 67) (27.3%)	Test statistics	*df*	*p* value
Duration of baseline episode (weeks)							
Median (IQR)	8 (6–20)	8 (4–20)	8 (4–16)	8 (8–20)	2.77^a^	2	0.25
Symptomatic remission, ever							
*n* (%)	149 (63.1)	53 (60.9)	55 (64.7)	41 (64.1)	0.30^b^	2	0.86
Symptomatic recovered, ever							
*n* (%)	63 (28.4)	25 (31.3)	21 (25.3)	17 (28.8)	0.64^b^	2	0.73
GAF symptoms change							
Mean (sd)	13.3 (26.8)	14.9 (27.1)	12.1 (25.1)	11.8 (30.6)	0.14^c^	95	0.87
GAF disability change							
Mean (sd)	9.4 (23.9)	8.6 (25.6)	6.7 (21.9)	16.8 (23.6)	1.12^c^	96	0.33

*w* weeks, *sd* standard deviation, *df* degrees of freedom, *GAF* global assessment of functioning

^a^Rank test *χ* ^2^ for the count data

^b^ *χ* ^2^ tests for categorical variables

^c^ANOVA test for continuous variables

### Pattern of care over the follow-up period

Patterns of care during the follow-up period after first contact with mental health services for psychosis by ethnicity are presented in Table 2. Excluding admissions on first contact with mental health services, 70% of our sample was re-admitted at least once, and 30% of our sample had ≥3 hospital re-admission during the follow-up period. Patients of Black Caribbean ethnicity had a shorter time to first re-admission after first contact (median_weeks_ = 46.2, IQR = 23.6–114.0) compared with Black African and White British ethnic groups (rank test *χ* ^2^ = 5.32, *df* = 2, *p* = 0.07). The Black Caribbean ethnic group had the longest (median_days_ = 141.0, IQR = 42.0–362.0), and patients of White British ethnicity had the shortest (median_days_ = 69.0, IQR = 38.0–173.0) overall length of time spent in psychiatric units; however, neither of these differences met the standard threshold for statistical significance. Further, a higher proportion of those of Black African and Black Caribbean ethnicity had compulsory re-admissions (*χ* ^2^ = 17.34, *p* = 0.002) and instances of police involvement during an admission to a psychiatric unit (*χ* ^2^ = 22.82, *p* < 0.001) compared with the White British ethnic group.

**Table 2:** Service utilisation over the first 5 years after first contact with mental health services for psychosis, by ethnicity

Service utilisation	Total (*n* = 245)	White British (*n* = 93) (38.0%)	Black African (*n* = 85) (34.7%)	Black Caribbean (*n* = 67) (27.3%)	Test statistics	*df*	*p* value
Time to first re-admission (weeks)							
Median (IQR)	50.1 (15.1–107.6)	51.4 (13.3–111.4)	52.1 (16.1–93.6)	46.2 (23.6–114.0)	5.32^a^	2	0.07
Admissions, *n* (%)							
None	71 (30.7)	30 (35.7)	21 (25.3)	20 (31.3)	4.89^b^	4	0.30
1–2	93 (40.3)	33 (39.3)	39 (47.0)	21 (32.8)			
>3	67 (29.0)	21 (25.0)	23 (27.7)	23 (35.9)			
Length of inpatient stay (days)							
Median (IQR)	107.0 (38.5–275.5)	69.0 (38.0–173.0)	122.5 (37.0–300.0)	141.0 (42.0–362.2)	4.93^a^	2	0.09
Compulsory, *n* (%)							
None	60 (34.3)	29 (46.8)	12 (19.1)	19 (38.0)	17.34^b^	4	0.002
1–2	80 (45.7)	26 (41.9)	38 (60.3)	16 (32.0)			
>3	35 (20.0)	7 (11.3)	13 (20.6)	15 (30.0)			
Police involved, *n* (%)							
None	74 (42.5)	35 (56.5)	14 (22.6)	25 (50.0)	22.82^b^	4	<0.001
1–2	79 (45.4)	24 (38.7)	40 (64.5)	15 (30.0)			
>3	21 (12.1)	3 (4.8)	8 (12.9)	10 (20.0)			
Community services							
Median (IQR)	3 (2–5)	3 (2–4)	3 (2–5)	3 (2–5.5)	3.19^a^	2	0.20

*w* weeks, *d* days, *IQR* inter-quartile range, *sd* standard deviation, *df* degrees of freedom

^a^Rank test *χ* ^2^ for the count data

^b^ *χ* ^2^ tests for categorical variables

### Socio-demographic characteristics over the follow-up period

By the end of the follow-up period, a higher proportion of Black Caribbean patients lived alone (61.2% of *n* = 67); while a substantial proportion of the Black African ethnic group (25.3% of *n* = 93) lived in supported accommodation (*χ* ^2^ = 10.88, *df* = 2, *p* = 0.03) as shown in Table 3. A lower proportion of White British patients were single (67% of *n* = 91) compared to those of the Black African (83.3% of *n* = 84) and Black Caribbean (81.8% of *n* = 66) ethnic groups (*χ* ^2^ = 7.81, *df* = 2, *p* = 0.02). Moreover, 26% (*n* = 17/66) of White British (compared to 8% (*n* = 6/72) of Black African and 5% (*n* = 3/56) of Black Caribbean ethnic groups) lived in privately rented accommodations; whereas, 93% (*n* = 52/56) of the Black Caribbean ethnic group were housed by local housing association services (*χ* ^2^ = 25.05, *df* = 4, *p* < 0.001).

**Table 3:** Socio-demographic characteristics by the follow-up period, by ethnicity

Demographics at follow-up	Total (*n* = 245)	White British (*n* = 93) (38.0%)	Black African (*n* = 85) (34.7%)	Black Caribbean (*n* = 67) (27.3%)	Test statistics	*df*	*p* value
Living arrangement, *n* (%)							
Alone	116 (47.9)	39 (42.4)	36 (43.4)	41 (61.2)	10.88^a^	4	0.03
Not alone	80 (33.1)	39 (42.4)	26 (31.3)	15 (22.4)			
Supported accommodation	46 (19.0)	14 (15.2)	21 (25.3)	11 (16.4)			
Relationship status, *n* (%)							
Single	185 (76.8)	61 (67.0)	70 (83.3)	54 (81.8)	7.81^a^	2	0.02
Stable relationship	56 (23.2)	30 (33.0)	14 (16.7)	12 (18.2)			
Employment, *n* (%)							
Unemployed	191 (81.6)	73 (86.9)	65 (76.5)	53 (81.5)	3.07^a^	2	0.22
Employed	43 (18.4)	11 (13.1)	20 (23.5)	12 (18.5)			
Type of accommodation, *n* (%)							
Owned	11 (5.7)	5 (7.6)	6 (8.3)	–	25.05^a^	6	<0.001
Housing association/Local authority rented	142 (73.2)	38 (57.6)	52 (72.2)	52 (92.9)			
Privately rented	26 (13.4)	17 (25.8)	6 (8.3)	3 (5.4)			
Homeless	15 (7.7)	6 (9.1)	8 (11.1)	1 (1.8)			

*df* degrees of freedom

^a^ *χ* ^2^ tests for categorical variables

## Discussion

We investigated the differences in the illness trajectories and pattern of care between White British, Black African and Black Caribbean ethnic groups during the 5-year of follow-up period. Our findings highlight that during the first 5 years of illness after first contact with mental health services, the longitudinal trajectory of psychosis in patients of Black ethnicity is characterised by longer inpatient stays, higher rates of compulsory admissions and increased instances of police involvement during or shortly before a re-admission to a psychiatric hospital compared with patients of White British ethnicity. This pattern of care identified for those in Black ethnic groups was not reflected in the overall functional disability and illness severity observed in their illness course, or their likelihood to meet the criteria for remission or recovery during the follow-up period.

### Methodological considerations

In the present study, we utilised a well-characterised sample of patients presenting for the first time with psychosis. Therefore, our sample represented a patient population that many UK clinicians see in everyday clinical practice, and our findings are not likely to be confounded by chronicity of illness or prolonged medication use [37]. By categorising those of Black African and Black Caribbean ethnicity into separate groups, we provide insights into illness trajectories that are specific to these ethnic populations. The measure of ethnicity that we employed in this study is highly reliable as it has previously shown a significant correlation with genetic ancestry derived using a panel of 57 ancestry informative genetic markers in the original GAP sample [38]. As the evidence suggests that first 5 years of illness constitute a critical period for determining longitudinal outcomes [39, 40], results reported here may have captured the most informative outcomes of illness progression across three major ethnic groups. Additionally, the overall drop-out rate in the present study was substantially lower than in many previous studies [8, 41, 42] with no evidence of attrition bias.

Our findings should be interpreted in light of methodological limitations. Generally, longitudinal studies tend to suffer from systematic bias due to non-random loss of information during the follow-up period. Nonetheless, in the present study considerable efforts have been made to minimise this potential bias by establishing the whereabouts, deaths and emigration status for 92% of our sample. One of the major limitations of the present study may be that the definition of the administrative remission was based solely on the electronic case notes as it might have been difficult to accurately and reliably define remission from notes, partly because there might not always have been information available on patients’ well-being when they were not in contact with mental health services, and partly because in some clinical notes might have been difficult to interpret. The quality and completeness of information reported in the clinical notes for each case inevitably varied, which in turn may have introduced bias. It is also possible that clinicians might not have always recorded in the electronic clinical records when symptoms were present and thus in some instances patients may have been classified inaccurately as remitted or recovered. Nonetheless, it has been shown that it is possible to reliably quantify the course of disorder using routine data from clinical notes [43]. Indeed, in the present study the rates of remission and recovery are consistent with earlier studies which collected data either from face-to-face interviews only [44] or extracted it retrospectively [45]. It is also feasible that some patients might have sought or purchased mental healthcare elsewhere, or sought alternative means to manage their symptoms, and thus would not have been registered in the SLaM electronic notes or included in the present study. Similarly, those patients who were reluctant to seek help would not be included in our sample; this in turn may reduce generalisability of our results. Further, we were unable to investigate whether the longitudinal outcomes differed depending on the immigrant generation to which our ethnic groups belong. Since female patients tend to have a less severe illness course [18], the small population of women in our sample may have increased the proportion of cases with a more severe illness course. Finally, even though the compared ethnic groups were not matched by age and sex, considerable efforts were made to ascertain a sample of patients who were representative of the FEP population in age, gender, ethnicity, educational qualifications, and employment status at the time of the study entry.

### Longitudinal course and outcome of first episode psychosis

In contrast to the findings from the AESOP-10 ethnicity study [46], we did not observe that patients of Black ethnicity had significantly elevated rates of hospital re-admissions over the 5-year period of follow-up compared with White British patients. Consistently with the AESOP-10 ethnicity study [46], our results highlighted that both Black African and Black Caribbean ethnic groups had longer total inpatient admissions than their White British counterparts. It may be argued that a longer duration of psychiatric hospital admission over the study period may be an indicator of a more severe illness course in patients of Black ethnicity. For example, in the AESOP-10 ethnicity study [46] it was found that patients of Black ethnicity were less likely to achieve remission and recovery compared to their White British counterparts. We did not find evidence to support this in our study, which may be due to methodological differences between our study and the AESOP-10 ethnicity study. Specifically, information at follow-up in the GAP-5 study was obtained from electronic case records only; whereas, follow-up data in the AESOP-10 ethnicity study were collected from both face-to-face interviews and case records. The length of follow-up in the present study was also shorter and the sample utilised in the final analysis was smaller compared to the AESOP-10 ethnicity study. The measures of clinical course between the studies were different; for example, in the GAP-5 study we focused on presence or absence of remission only, whereas in the AESOP-10 ethnicity paper the information on the three course types was also reported. The selection of patients at the time of first recruitment between the studies was also different [21, 46]. Nonetheless, it is equally plausible that patients’ living arrangements were important contributing factors to the longer inpatient stays among the patients of Black ethnicity observed in our study [47]. Indeed, a higher proportion of patients of Black ethnicity lived alone, was single or was housed by local authorities compared with their White British counterparts. This may suggest that longer inpatient stays may have been required due to less easily accessed accommodation after hospital discharges. Although some have raised a cause for concern that ethnic minority patients under utilise psychiatric community services after contact with mental health services [8], our results showed that this was not the case for Black African and Black Caribbean ethnic groups when compared to White British counterparts during follow-up.

Previously, it has been reported that people of Black ethnicity were more likely to be compulsorily detained compared with patients of White ethnicity during 1 year [48] and 2 years of follow-up [8]. Our results showed that this still remains the case during the first 5 years of illness after first contact with mental health services for psychosis. It has been suggested that the risk for compulsory detentions is amplified by a reluctance to seek help during a mental health crisis among those of Black African [48, 49] and Black Caribbean [50] ethnicity, potentially increasing the need for admissions under MHA legislation. The alleged unwillingness to utilise the available services at the time of mental health crisis has been linked to a variety of factors including distrust of psychiatric services [51], lack of insight into mental health difficulties [52] and language barriers [49]. There may also be important cultural factors to consider. For example, it has been shown that persecutory beliefs, and hallucinatory experiences, especially those of religious content, may be culturally acceptable among individuals of Black Caribbean ethnicity [53] and as such they may not have the same clinical significance as in White British counterparts potentially contributing to delays in presentation, and increasing the likelihood of MHA utilisation during the mental health crisis [54]. Nonetheless, there remains a paucity of research into cultural aspects that may explain the differences in pattern of care received by patients of Black ethnicity. Overall, our findings suggest that the factors which led to a higher rate of compulsory admissions among Black individuals in the past have not yet diminished. Further, patients of Black African ethnicity tended to have multiple instances of police involvements during hospital re-admissions. It has previously been shown that family members of those of Black ethnicity contact the police more frequently at times of clinical deterioration in their relative [50]; though we were unable to test if this was a factor in the increased rates of police involvements during hospital re-admissions in Black cases.

Additionally, we found that the proportion of unemployed increased in White British and Black African ethnic groups by the end of the follow-up period. While it is common for individuals with psychosis to struggle to develop or maintain stable relationships [55], there was an increased proportion of single individuals in the Black African and Black Caribbean ethnic groups compared with the White British group. Cumulatively these findings suggest that patients of Black ethnicity become increasingly socially excluded as their illness progresses. These findings mirror the AESOP-10 ethnicity study [46] which highlighted that social disadvantage and isolation persist beyond the 5-year period after the first contact mental health services.

## Conclusion

Our findings are in accord with those obtained in the AESOP-10 ethnicity study and demonstrate that clinical outcomes are not better among black Caribbean and black African compared to white British patients. Differences remain in patterns of care among those of Black African, Black Caribbean and White British ethnicity resident in London during the first 5 years after first contact with mental health services for psychosis. The longitudinal trajectory of psychosis in patients of Black ethnicity is characterised by longer inpatient stays, higher rates of compulsory admissions and increased instances of police involvement during or shortly before a re-admission to a psychiatric hospital compared with patients of White British ethnicity. The observed pattern of care in Black ethnic groups was not explained by increased functional disability and illness severity or related to differing remission or recovery rates during the follow-up period. The prognosis remains poor in terms of social functioning among Black ethnic groups. Further study is required to establish whether these differences reflect social or clinical differences between ethnic groups. Nonetheless, our findings reiterate a greater need for action in health systems and social policy to challenge and reduce these disparities.

## Electronic supplementary material

Below is the link to the electronic supplementary material.

Supplementary material 1 (DOCX 48 kb)
