# Supplementary material for: Patterns of illness and care over the 5 years following onset of psychosis in different ethnic groups; the GAP-5 study
Source: Soc Psychiatry Psychiatr Epidemiol. 2017 Jul 5;52(9):1101–11. doi: 10.1007/s00127-017-1417-6 (PMC5581822; doi:10.1007/s00127-017-1417-6)
Supplement: Supplementary file 1 — Supplementary material 1 (DOCX 48 kb) [file 127_2017_1417_MOESM1_ESM.docx]

**Supplementary Table 1.** Baseline diagnosis, socio-demographic and clinical characteristics, by ethnicity

| Baseline socio-demographic and clinical characteristics | | | Total  *n*=297 | White British  *n*=111 (37.4) | Black African  *n*=110 (37.0) | Black Caribbean  *n*=76 (25.6) | Statistics | df | *p*-value |
| --- | --- | --- | --- | --- | --- | --- | --- | --- | --- |
|  |  | |  |  |  |  |  |  |  |
| Age _years_ , Mean (s.d.) | | | 28.3 (8.8) | 29.5 (9.9) | 26.8 (7.5) | 28.6 (8.6) | F=2.51 | 284 | 0.08 |
|  |  | |  |  |  |  |  |  |  |
| DUP _days_ , Mean (s.d.) | | | 39.2 (126.3) | 41.6 (148.4) | 36.8 (103.7) | 40.2 (131.8) | F=0.02^a^ | 173 | 0.98 |
|  |  | |  |  |  |  |  |  |  |
| Gender, *n* (%) | | |  |  |  |  |  |  |  |
|  | Women | | 100 (34.6) | 34 (31.5) | 34 (31.8) | 32 (43.2) | *x*^2^=3.28 | 2 | 0.19 |
|  | Men | | 189 (65.4) | 74 (68.5) | 73 (68.2) | 42 (56.8) |  |  |  |
|  |  | |  |  |  |  |  |  |  |
| Diagnosis, *n* (%) | | |  |  |  |  |  |  |  |
|  | Non-affective psychosis | | 185 (75.2) | 63 (71.6) | 71 (75.5) | 51 (79.7) | *x*^2^=1.31 | 2 | 0.52 |
|  | Affective psychosis | | 61 (24.8) | 25 (28.4) | 23 (24.5) | 13 (20.3) |  |  |  |
|  |  | |  |  |  |  |  |  |  |
| Living arrangements, *n* (%) | | |  |  |  |  |  |  |  |
|  | Alone | | 69 (39.4) | 26 (37.1) | 23 (32.4) | 20 (58.8) | *x*^2^=6.98 | 2 | 0.03 |
|  | With partner or parents | | 106 (60.6) | 44 (62.9) | 48 (67.6) | 14 (41.2) |  |  |  |
|  |  | |  |  |  |  |  |  |  |
| Relationship status, *n* (%) | | |  |  |  |  |  |  |  |
|  | Single | | 130 (74.3) | 52 (74.3) | 52 (73.2) | 26 (76.5) | *x*^2^=0.13 | 2 | 0.94 |
|  | Stable relationship | | 45 (25.7) | 18 (25.7) | 19 (26.8) | 8 (23.5) |  |  |  |
|  |  | |  |  |  |  |  |  |  |
| Employment, *n* (%) | | |  |  |  |  |  |  |  |
|  | | Unemployed | 114 (66.3) | 46 (68.7) | 40 (56.3) | 28 (82.4) | *x*^2^=7.24 | 2 | 0.03 |
|  | | Employed | 58 (33.7) | 21 (31.3) | 31 (43.7) | 6 (17.6) |  |  |  |
|  | |  |  |  |  |  |  |  |  |
| GAF symptoms, Mean (s.d.) | | | 46.7 (20.1) | 47.1 (21.0) | 47.6 (18.9) | 43.6 (21.2) | F=0.31 | 119 | 0.73 |
|  | |  |  |  |  |  |  |  |  |
| GAF disability, Mean (s.d.) | | | 55.1 (18.3) | 56.1 (19.9) | 57.0 (16.2) | 48.1 (18.2) | F=1.91 | 119 | 0.15 |

s.d., standard deviations; DUP, duration of untreated psychosis; d.f., degrees of freedom; GAF, global assessment of functioning

^a^ the results are presented after log-transformation

**Supplementary Table 2.** Baseline demographic characteristics by administrative outcome

| Baseline demographic characteristics | | Followed up  *n*=245 (84.5%) | Unable to trace  *n*=23 (7.9%) | Abroad  *n*=12 (4.1%) | Died  *n*=4 (1.4%) | Moved  *n*=6 (2.1%) | Statistics | df | *p*-value |
| --- | --- | --- | --- | --- | --- | --- | --- | --- | --- |
|  |  |  |  |  |  |  |  |  |  |
| Gender, *n* (%) | |  |  |  |  |  |  |  |  |
|  | Female | 82 (34.2) | 7 (30.4) | 7 (58.3) | 2 (50.0) | 1 (16.7) | *x*^2^=4.44 | 4 | 0.35 |
|  | Male | 158 (65.8) | 16 (69.6) | 5 (41.7) | 2 (50.0) | 5 (83.3) |  |  |  |
|  |  |  |  |  |  |  |  |  |  |
| Age at first contact, *n* (%) | | 27.9 (8.1) | 29.1 (11.7) | 26.3 (5.9) | 44.5 (18.9) | 29.8 (4.7) | F=4.05 | 282 | 0.003 |
|  |  |  |  |  |  |  |  |  |  |
| Ethnicity, *n* (%) | |  |  |  |  |  |  |  |  |
|  | White British | 93 (38.0) | 9 (39.1) | 1 (8.3) | 2 (50.0) | 3 (50.0) | *x*^2^=18.36 | 8 | 0.02 |
|  | Black African | 84 (34.7) | 10 (43.5) | 11 (91.7) | 1 (25.0) | 1 (16.7) |  |  |  |
|  | Black Caribbean | 67 (27.3) | 4 (17.4) | - | 1 (25.0) | 2 (33.3) |  |  |  |
|  |  |  |  |  |  |  |  |  |  |
| Living arrangements, *n* (%) | |  |  |  |  |  |  |  |  |
|  | Alone | 61 (41.5) | 2 (15.4) | 3 (37.5) | 1 (33.3) | 2 (50.0) | *x*^2^=3.66 | 4 | 0.45 |
|  | With partner or parents | 86 (58.5) | 11 (84.6) | 5 (62.5) | 2 (66.7) | 2 (50.0) |  |  |  |
|  |  |  |  |  |  |  |  |  |  |
| Relationship status, *n* (%) | |  |  |  |  |  |  |  |  |
|  | Single | 107 (72.8) | 9 (69.2) | 8 (100.0) | 2 (66.7) | 4 (100.0) | *x*^2^=4.59 | 4 | 0.33 |
|  | Stable relationship | 40 (27.2) | 4 (30.8) | - | 1 (33.3) | - |  |  |  |

s.d., standard deviations; d.f., degrees of freedom

**Supplementary Table 3.** Baseline demographic characteristics for those who were lost to follow up compared to individuals with full follow up data

| Baseline sample characteristics | | Lost to follow up  *n*=45 (15.5%) | Followed up  *n*=245 (84.5%) | Statistics | df | *p*-value |
| --- | --- | --- | --- | --- | --- | --- |
|  |  |  |  |  |  |  |
| Age _years_, Mean (s.d.) | | 30.1 (11.5) | 27.9 (8.1) | t=-1.46 | 281 | 0.15 |
|  |  |  |  |  |  |  |
| Gender, *n* (%) | |  |  |  |  |  |
|  | Women | 17 (37.8) | 82 (34.2) | *x*^2^=0.22 | 1 | 0.64 |
|  | Men | 28 (62.2) | 158 (65.8) |  |  |  |
|  |  |  |  |  |  |  |
| Ethnicity, *n* (%) | |  |  |  |  |  |
|  | White British | 15 (33.3) | 93 (38.0) | *x*^2^=5.04 | 2 | 0.08 |
|  | Black African | 23 (51.1) | 85 (34.7) |  |  |  |
|  | Black Caribbean | 7 (15.6) | 67 (27.3) |  |  |  |
|  |  |  |  |  |  |  |
| Living arrangements, *n* (%) | |  |  |  |  |  |
|  | Alone | 8 (28.6) | 61 (41.5) | *x*^2^=1.65 | 1 | 0.20 |
|  | With partner or parents | 20 (71.4) | 86 (58.5) |  |  |  |
|  |  |  |  |  |  |  |
| Relationship status, *n* (%) | |  |  |  |  |  |
|  | Single | 23 (82.1) | 107 (72.8) | *x*^2^=1.08 | 1 | 0.30 |
|  | Stable relationship | 5 (17.9) | 40 (27.2) |  |  |  |
|  |  |  |  |  |  |  |
| GAF symptoms, Mean (s.d.) | | 41.5 (17.0) | 47.7 (20.6) | t=1.24 | 118 | 0.22 |
|  |  |  |  |  |  |  |
| GAF disability, Mean (s.d.) | | 54.7 (18.4) | 55.1 (18.4) | t=0.09 | 118 | 0.93 |

s.d., standard deviations; d.f., degrees of freedom; GAF, global assessment of functioning

**Supplementary Table 4.** Demographic characteristics by ethnicity at 5 years follow up

| Demographic characteristics | | Total  *n*=245 | White British  *n*=93 (38.0%) | Black African  *n*=85 (34.7%) | Black Caribbean  *n*=67 (27.3%) | Test statistics | df | *p*-value |
| --- | --- | --- | --- | --- | --- | --- | --- | --- |
|  |  |  |  |  |  |  |  |  |
| Follow up, Mean (s.d.) | | 5.1 (2.4) | 4.9 (2.4) | 4.9 (2.2) | 5.6 (2.6) | F=2.12 | 243 | 0.12 |
|  |  |  |  |  |  |  |  |  |
| Gender, *n* (%) | |  |  |  |  |  |  |  |
|  | Women | 82 (34.2) | 29 (31.5) | 23 (28.1) | 30 (45.5) | *x*^2^=5.39 | 2 | 0.07 |
|  | Men | 158 (65.8) | 63 (68.5) | 59 (71.9) | 36 (54.5) |  |  |  |

s.d., standard deviation; d.f., degrees of freedom

**Supplementary Table 5.** Clinical presentation, pattern of care over the follow up period and socio demographic characteristics at follow up by the diagnoses

| Clinical presentation, pattern of care over the follow up period and socio-demographic characteristics at follow up | | Measure unites | Non-affective psychosis  *n*=162 (74.6%) | Affective psychosis  *n*=55 (25.4%) | Statistics | df | *p*-value |
| --- | --- | --- | --- | --- | --- | --- | --- |
| *Clinical presentation* | |  |  |  |  |  |  |
|  | Duration of baseline episode, w | Median (IQR) | 8 (6-20) | 8 (4-16) | z=1.10 |  | 0.27 |
|  | Symptomatic remission, ever | *n* (%) | 94 (58.8) | 40 (76.9) | *x*^2^=5.57 | 1 | 0.02 |
|  | Symptomatic recovered, ever | *n* (%) | 46 (28.8) | 13 (24.5) | *x*^2^=0.35 | 1 | 0.55 |
|  | GAF symptoms change | Mean (sd) | 16.4 (16.8) | 12.1 (23.7) | t=0.72 | 81 | 0.47 |
|  | GAF disability change | Mean (sd) | 9.3 (25.0) | 10.4 (23.8) | t=0.85 | 82 | 0.85 |
|  |  |  |  |  |  |  |  |
| *Service utilisation* | |  |  |  |  |  |  |
|  | Time to first readmission, w | Median (IQR) | 57.8 (21.7-122.0) | 35.1 (15.3-96.0) | z=0.99 |  | 0.32 |
|  |  |  |  |  |  |  |  |
|  | Admissions | *n* (%) |  |  | *x*^2^=8.81 | 2 | 0.01 |
|  | None |  | 39 (24.8) | 22 (44.0) |  |  |  |
|  | 1-2 |  | 74 (47.1) | 13 (26.0) |  |  |  |
|  | >3 |  | 44 (28.3) | 15 (30.0) |  |  |  |
|  |  |  |  |  |  |  |  |
|  | Length of inpatient stay, d | Median (IQR) | 110 (39-288) | 48 (38-140) | z=1.68 |  | 0.09 |
|  |  |  |  |  |  |  |  |
|  | Compulsory | *n* (%) |  |  | *x*^2^=0.12 | 2 | 0.94 |
|  | None |  | 40 (32.3) | 11 (32.4) |  |  |  |
|  | 1-2 |  | 59 (47.6) | 17 (50.0) |  |  |  |
|  | >3 |  | 25 (20.2) | 6 (17.6) |  |  |  |
|  |  |  |  |  |  |  |  |
|  | Police involved | *n* (%) |  |  | *x*^2^=1.67 | 2 | 0.43 |
|  | None |  | 54 (43.9) | 11 (32.4) |  |  |  |
|  | 1-2 |  | 54 (43.9) | 17 (50.0) |  |  |  |
|  | >3 |  | 15 (12.2) | 6 (17.7) |  |  |  |
|  |  |  |  |  |  |  |  |
|  | Community services | Median (IQR) | 3 (1-3) | 3 (2-3) | *x*^2^=-1.70 |  | 0.09 |
|  |  |  |  |  |  |  |  |
| *Demographics at follow up* | |  |  |  |  |  |  |
|  | Living arrangement | *n* (%) |  |  | *x*^2^=11.29 | 2 | 0.004 |
|  | Alone |  | 79 (49.7) | 22 (40.0) |  |  |  |
|  | Not alone |  | 44 (27.7) | 28 (50.9) |  |  |  |
|  | Supported accommodation |  | 36 (22.6) | 5 (9.1) |  |  |  |
|  |  |  |  |  |  |  |  |
|  | Relationship status | *n* (%) |  |  | *x*^2^=7.60 | 1 | 0.006 |
|  | Single |  | 130 (81.8) | 35 (63.6) |  |  |  |
|  | Stable relationship |  | 29 (18.2) | 20 (36.4) |  |  |  |
|  |  |  |  |  |  |  |  |
|  | Employment | *n* (%) |  |  | *x*^2^=0.01 | 1 | 0.94 |
|  | Unemployed |  | 127 (80.9) | 41 (80.4) |  |  |  |
|  | Employed |  | 30 (19.1) | 10 (19.6) |  |  |  |
|  |  |  |  |  |  |  |  |
|  | Type of accommodation | *n* (%) |  |  | *x*^2^=8.36 | 3 | 0.04 |
|  | Owned |  | 6 (4.6) | 3 (7.7) |  |  |  |
|  | Housing association/Local authority rented |  | 100 (76.3) | 25 (64.1) |  |  |  |
|  | Privately rented |  | 13 (9.9) | 10 (25.6) |  |  |  |
|  | Homeless |  | 12 (9.2) | 1 (2.6) |  |  |  |

w, weeks; d, days; s.d., standard deviation; d.f., degrees of freedom; GAF, global assessment of functioning
